# Novel Surrogate Markers of Cardiovascular Risk in the Setting of Autoimmune Rheumatic Diseases: Current Data and Implications for the Future

**DOI:** 10.3389/fmed.2022.820263

**Published:** 2022-06-30

**Authors:** Anna Mandel, Andreas Schwarting, Lorenzo Cavagna, Konstantinos Triantafyllias

**Affiliations:** ^1^Department of Internal Medicine I, Division of Rheumatology and Clinical Immunology, Johannes Gutenberg University Medical Center, Mainz, Germany; ^2^Department of Rheumatology, Rheumatology Center RL-P, Bad Kreuznach, Germany; ^3^Division of Rheumatology, University and IRCCS Policlinico S. Matteo Foundation, Pavia, Italy

**Keywords:** surrogate marker, cardiovascular risk, autoimmune, rheumatic disease, prevention

## Abstract

Patients suffering from rheumatologic diseases are known to have an increased risk for cardiovascular disease (CVD). Although the pathological mechanisms behind this excess risk have been increasingly better understood, there still seems to be a general lack of consensus in early detection and treatment of endothelial dysfunction and CVD risk in patients suffering from rheumatologic diseases and in particular in those who haven't yet shown symptoms of CVD. Traditional CVD prediction scores, such as Systematic Coronary Risk Evaluation (SCORE), Framingham, or PROCAM Score have been proposed as valid assessment tools of CVD risk in the general population. However, these risk calculators developed for the general population do not factor in the effect of the inflammatory burden, as well as other factors that can increase CVD risk in patients with rheumatic diseases, such as glucocorticoid therapy, abnormal lipoprotein function, endothelial dysfunction or accelerated atherosclerosis. Thus, their sole use could lead to underestimation of CVD risk in patients with rheumatic diseases. Therefore, there is a need for new biomarkers which will allow a valid and early assessment of CVD risk. In recent years, different research groups, including ours, have examined the value of different CVD risk factors such as carotid sonography, carotid-femoral pulse wave velocity, flow-mediated arterial dilation and others in the assessment of CVD risk. Moreover, various novel CVD laboratory markers have been examined in the setting of autoimmune diseases, such as Paraoxonase activity, Endocan and Osteoprotegerin. Dyslipidemia in rheumatoid arthritis (RA) is for instance better quantified by lipoproteins and apolipoproteins than by cholesterol levels; screening as well as pre-emptive carotid sonography hold promise to identify patients earlier, when prophylaxis is more likely to be effective. The early detection of subtle changes indicating CVD in asymptomatic patients has been facilitated through improved imaging methods; the inclusion of artificial intelligence (AI) shows promising results in more recent studies. Even though the pathophysiology of coronary artery disease in patients with autoimmune rheumatic diseases has been examined in multiple studies, as we continuously gain an increased understanding of this comorbidity, particularly in subclinical cases we still seem to fail in the stratification of who really is at risk—and who is not. A the time being, a multipronged and personalized approach of screening patients for traditional CVD risk factors, integrating modern imaging and further CV diagnostic tools and optimizing treatment seems to be a solid approach. There is promising research on novel biomarkers, likewise, methods using artificial intelligence in imaging provide encouraging data indicating possibilities of risk stratification that might become gold standard in the near future. The present review concentrates on showcasing the newest findings concerning CVD risk in patients with rheumatologic diseases and aims to evaluate screening methods in order to optimize CVD risk evaluation and thus avoiding underdiagnosis and undertreatment, as well as highlighting which patient groups are most at risk.

## Introduction

Cardiovascular disease (CVD) is one of the predominant causes of death and reduced quality of life worldwide (1). Half a century ago, traditional risk factors, such as systemic hypertension, physical inactivity, obesity, diabetes, smoking, and hypercholesterolemia have been described and then complemented by non-traditional risk factors, such as inflammation and consecutive atherosclerosis associated with RA and other autoimmune processes. Additional mechanisms linking RA and CVD include shared post translational modification of both peptides and proteins, and a multitude of subsequent immune responses, alterations in the composition and function of lipoproteins, increased oxidative stress, and endothelial dysfunction (2, 3). While the first mentioned are already broadly used for screening and diagnostics in cases with symptomatic vasculitis or corresponding genetic predisposition, and complex polygenetic risk, there seems to be a general lack of consensus in early detection and treatment of endothelial dysfunction and cardiovascular (CV) risk in patients suffering from rheumatologic diseases who haven't yet shown CVD symptoms. However, it is clearly established that CVD is between the leading comorbidities and the most common death causes in patients with autoimmune rheumatologic disorders. In fact, these patients had an increased 10-years risk of major adverse CV events like sudden cardiac death or ischemic stroke, regardless of the prior presence of a coronary artery disease. Additionally, the risk rises significantly for patients with RA and already persisting coronary artery disease (CAD) (4). Atherosclerosis might be directly mediated also by underlying autoimmune processes in patients with rheumatoid arthritis (5). Furthermore, it is expected that a part of the two-fold higher risk of heart failure and total mortality in RA may be due to myocardial disease associated with inflammation including elevated acute phase proteins, T-Cell subsets, proinflammatory cytokines and the presence of circulating auto-antibodies (5). Autoimmune rheumatic diseases are known to affect the valves, myocardium, pericardium as well as the cardiac vasculature and conduction system, leading to multiple cardiovascular manifestations that in some cases can remain clinically silent or lead to a considerable cardiovascular mortality and morbidity (6–10). Atherosclerosis plays a substantial role in CVD morbidity and mortality; the degree of coronary atherosclerosis observed in patients with rheumatic diseases can be as accelerated, diffuse, and extensive as in patients with diabetes mellitus (11). Although this high risk of CVD has been known for decades, patients with rheumatologic diseases generally receive poorer primary and secondary CVD preventive care than other high-risk patients.

**Figure 1:**
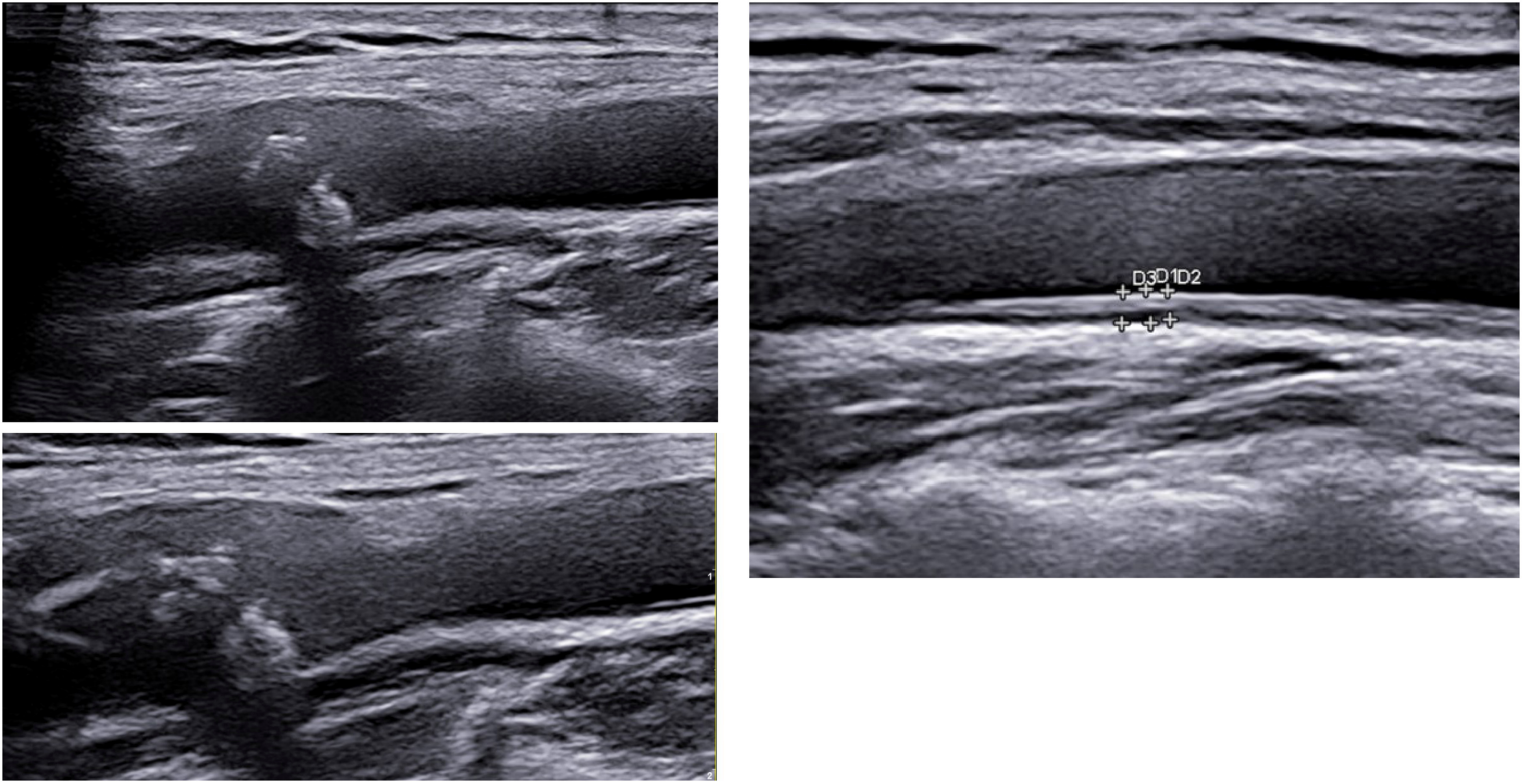
Carotid ultrasound imaging with plaque (left) and increased carotid intima media thickness (above) in two different patients, courtesy by Dr. Konstantinos Triantafyllias, Rheumatology center in Bad Kreuznach, Germany.

In 2009, the European League Against Rheumatism (EULAR) recommended screening, identification of CVD risk factors and CVD risk management based on expert opinion and since has published an update based on a growing body of evidence. One of the overarching principles that have been defined is that the rheumatologist is responsible for risk management in patients with inflammatory joint diseases. For patients with RA, ankylosing spondylitis and psoriatic arthritis, CVD risk assessment is recommended at least once every 5 years and should be reconsidered following major changes in antirheumatic therapy. Other recommendations include optimizing disease activity control, lifestyle recommendations and screening for asymptomatic atherosclerotic plaques by use of carotid ultrasound among others (12, 13) (Figure 1). More recently, Drosos et al. (14) published EULAR recommendations for patients affected by gout, vasculitis, systemic sclerosis, myositis, mixed connective tissue disease, Sjögren's syndrome, systemic lupus erythematosus, and antiphospholipid syndrome. The authors put an emphasis on the importance of regular screening and management of modifiable CVR factors. Several recommendations relied on expert opinion because high-quality evidence is scarce. Due to lack of validated rheumatic diseases-specific tools, they recommend the use of generic CVR prediction tools.

The consensus, however, is that in order to evaluate CVD risk in patients with rheumatic diseases, there is an inherent need for screening methods tailored to this specific patient group. Thus, as there is a lack of high-quality evidence, more studies are needed addressing this matter.

This review's aim is to give an overview over new advancements in the field of CVD risk assessment in patients with rheumatic diseases. In particular, we want to showcase novel approaches in the field of imaging technology and biomarkers, as well as highlighting the role of established methods. This could help to facilitate earlier diagnosis and treatment, thus preventing CV events and lead to a better outcome for these patients.

## CVD Surrogate Markers

### Arterial Stiffness: Measurements by Pulse Wave Velocity and Augmentation Index

**Figure 2:**
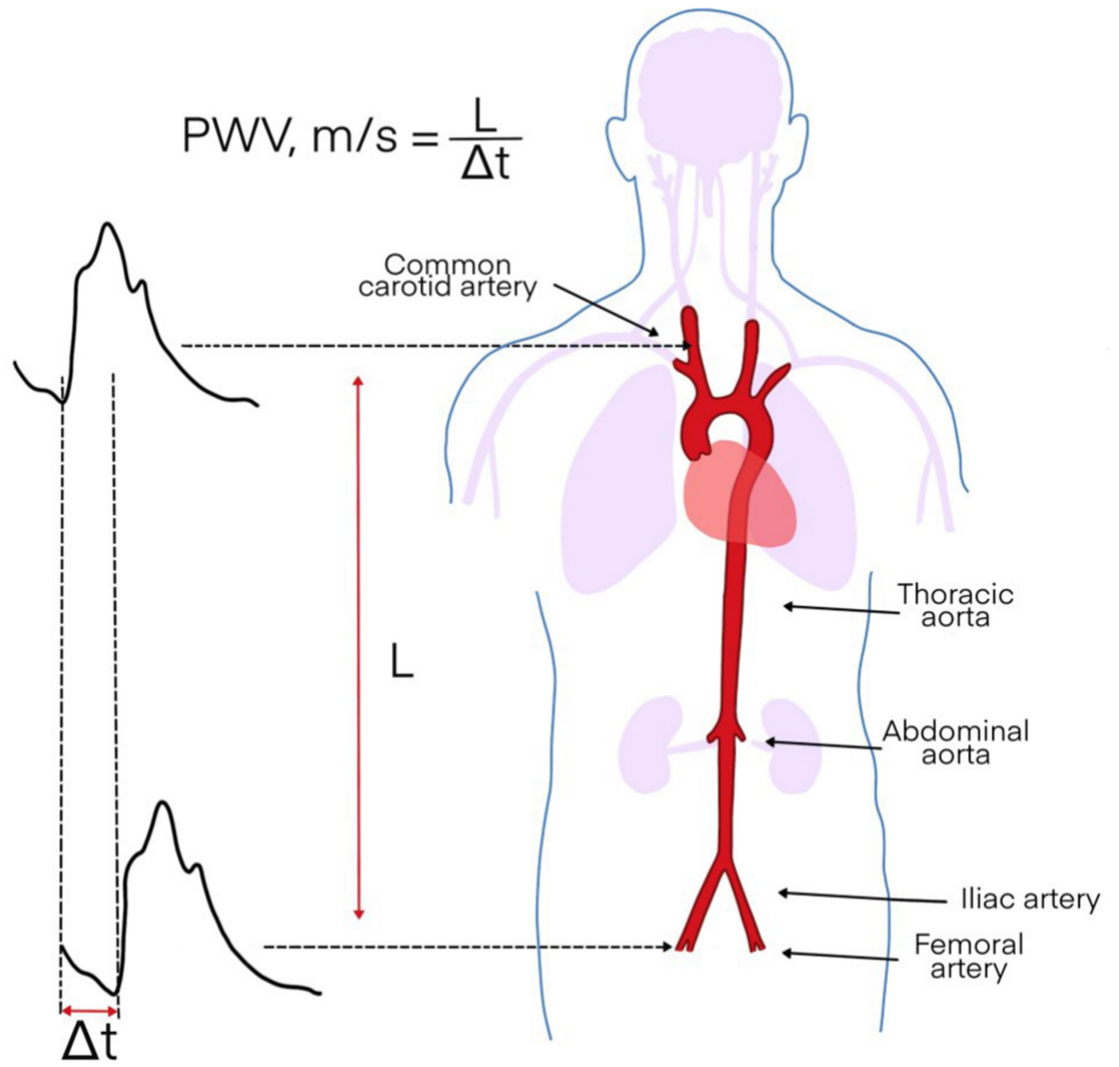
Principles of assessment of pulse wave velocity (PWV).

Pulse wave velocity (PWV) (Figure 2) has long been established as the gold standard for the assessment of aortic stiffness (AS) and is widely used for CV risk stratification; recent studies have shown that aortic stiffness measured through PWV has an independent predictive value for CV events in multiple populations, thus heightening its diagnostic value (15, 16). Increased arterial stiffness leads to diastolic dysfunction, which is the main responsible mechanism of heart failure in chronic inflammatory rheumatic diseases. Rheumatoid arthritis has long been characterized as a systemic disease with a well-defined high atherosclerotic burden. It has been shown that PWV is increased in these patients and that there is an association with age, disease duration, and erythrocyte sedimentation rate (ESR) (17). Our research group could examine PWV during the last few years in patients with various autoimmune rheumatic diseases, such as rheumatoid arthritis (8), mixed connective tissue disease (MCTD) (7), systemic lupus erythematosus (SLE) (10), and antisynthetase syndrome (ASyS) (11). In the case of MCTD and ASyS, aortic PWV was statistically significantly higher in comparison to respective control groups even after adjustment for possible confounding factors. Thus, a higher CVD risk could be postulated. Moreover, we could find that PWV and carotid sonography could improve screening of CV and cerebrovascular risk in patients with ASyS by identifying high risk patients who could have been missed by taking into account only traditional CVD risk factors. Interestingly, there was no difference in cfPWV of patients with SLE and healthy controls in our study. However, we found an independent statistically significant inverse association between estimated glomerular filtration rate (eGFR) and cfPWV in an SLE population with a widely normally ranged eGFR (10). Patients with fibromyalgia, a disease which belongs to the so called rheumatologic chronic pain syndromes and does not have a proven autoimmune background (18), showed higher AS than healthy controls (9). Another widely used indicator for CVD risk is the augmentation index (AIx), which is a measurement of peripheral arterial wave reflections. Although both PWV and AIx deliver information on aortic stiffness, they cannot be used interchangeably: Sakura et al. (19) investigated the relationship between aortic AIx and PWV by measuring them directly using a catheter and found no significant relationship between AI and PWV. The data of the Anglo-Cardiff Collaborative Trial suggested that the AIx might be more sensitive as a marker of arterial stiffening and risk in younger individuals, whereas PWV might be better suited for older individuals (20). PWV is still considered the gold standard method to measure arterial stiffness (21) and is widely used in the scientific community.

Still, there is a large number of studies that provided evidence that PWV, as well as AIx, are predictive for CV events and all-cause mortality in asymptomatic populations (22–24). Nowadays, devices are reasonably portable, relatively easy to use, time-efficient and non-invasive. Elliot et al. (25) reported acceptable to excellent PWV measurement accuracy by a novice operator following as little as 14 practice participants. However, both methods are still not routinely used in daily clinical practice.

## Cardiovascular Imaging: Detecting Subtle Changes

**Figure 3:**
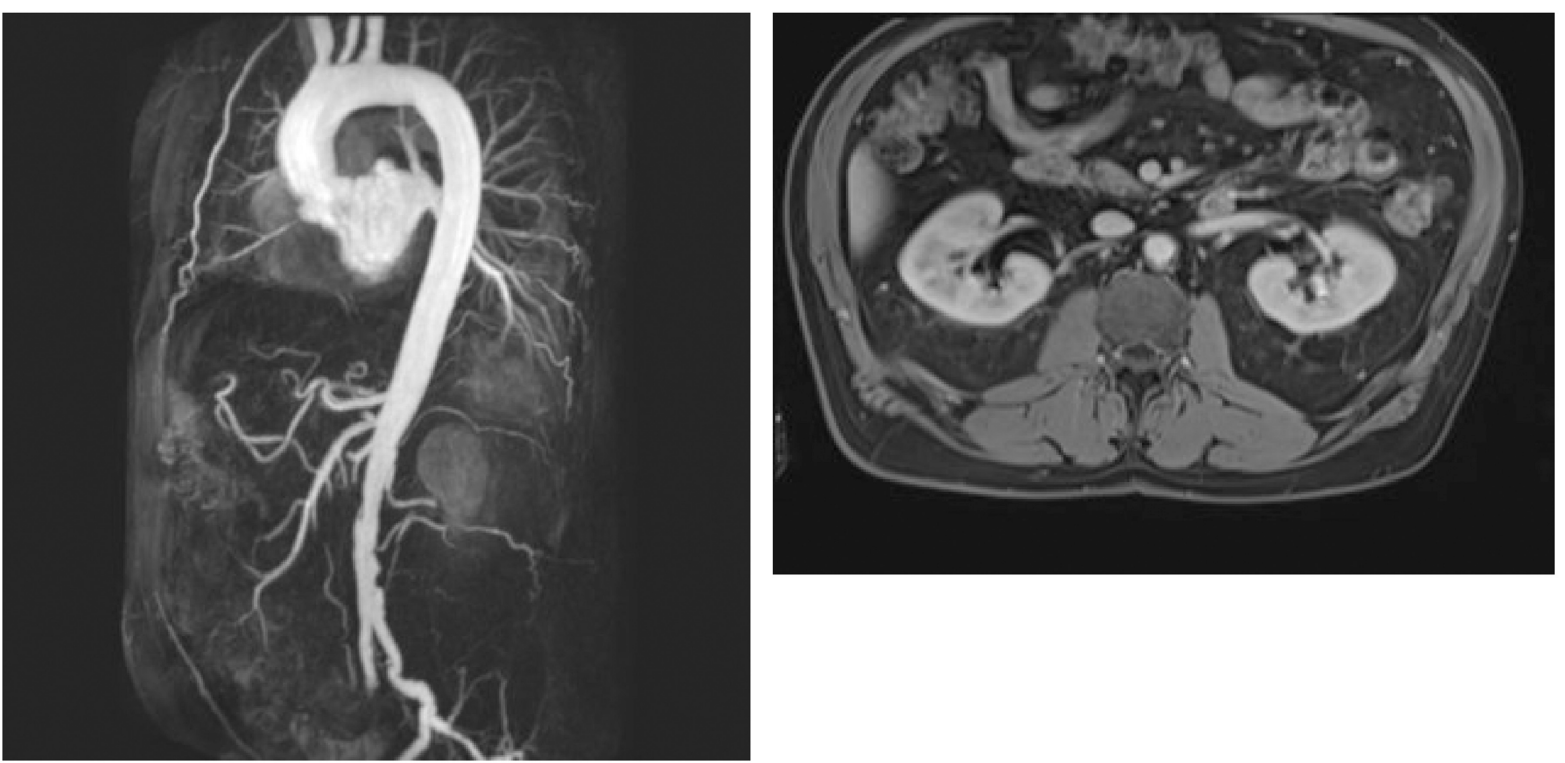
MIP reconstruction of a CE-MRA of a 64 years old patient's aorta and transversal T1 vibe dixon with excentric plaque of the aorta. Pictures by courtesy of Dr. med. Corinna Schorn, Rheumatology center in Bad Kreuznach, Germany.

Other non-invasive cardiovascular imaging modalities such as magnetic resonance imaging (26) (Figure 3), positron emission tomography (27), computed tomography (28), optical coherence tomography (29), and ultrasound (30) can be used for risk assessment and early detection of CVD in asymptomatic patients. These methods offer a variety of unique information concerning the morphological variations of atherosclerosis and differ in availability, practicability, and cost. MRI can assess plaque composition, such as calcification, lipid-rich necrotic core, and the thickness of the fibrotic cap (31). Cardiac Magnetic Resonance Imaging (cMRI) has been shown to detect myocardial abnormalities in RA patients without known cardiac disease (32). CMRI was also used in a comparison of myocardial structure and function in a cohort study of patients with RA with matched controls. Interestingly, mean left-ventricular mass was 26 g lower for the RA group compared to controls (*p* < 0.001), suggesting that the progression to heart failure in RA patients might be due to reduced myocardial mass rather than hypertrophy (33). Mavrogeni et al. (34) could show that cMRI was able to detect cardiac lesions in symptomatic patients with connective tissue disease and a normal echocardiography.

CT is mostly used to evaluate the degree of carotid artery stenosis, while F-fludeoxyglucose–positron emission tomography (FDG-PET) delivers vital information on the inflammation present in carotid atherosclerotic plaque. Ultrasound, specifically of the carotid arteries, has emerged as a widely available, relatively low-cost imaging method that has been established in preventative care in clinical settings. Carotid intima-media thickness (cIMT) and the presence of plaque have been used as surrogate markers for CVD risk in multiple studies. RA patients display a high prevalence of increased cIMT and carotid plaque (18); similarly, associations have been found with systemic sclerosis (35), ASyS (11), SLE, and many other inflammatory diseases. Interestingly, Ajeganova et al. showed in a 10-year case-control-study, that SLE patients had a three- to four-fold higher risk of CV events and death, compared with persons who do not have SLE but with a similar pattern of traditional CVD risk factors and subclinical atherosclerosis measured with carotid intima-media thickness and presence of carotid plaque (36). A significantly improved prediction of the adverse outcome could be accomplished with the combination of cIMT measures with the Systemic Lupus International Collaborating Clinics (SLICC) Damage Index and coexistence of SLE-antiphospholipid syndrome (SLE-APS) (37). These findings highlight the necessity of a comprehensive approach to risk stratification and management. Echocardiography has found its way into routine preventative care in patients with autoimmune and rheumatological diseases, as the presence of cardiac abnormalities, such as heart muscle damage, pericardial involvement and valvular heart disease are relatively common. However, early structural, and functional changes are often subtle and not easy to detect (37). Therefore, conventional echocardiographic parameters may often be not sufficient to find these early abnormalities of cardiac dysfunction especially if the global ventricular function is normal. Hence, the use of echocardiographic techniques such as tissue velocity imaging (TVI), ventricular strain imaging (SI), and strain rate imaging (SRI) can be useful for analysis of regional and longitudinal myocardial function (38).

Cardiovascular magnetic resonance imaging holds a promising role in the early detection of numerous pathophysiologic phenomena in subclinical patient populations (39). It allows for the evaluation of cardiac function, the identification of various disease entities such as myocardial oedema and inflammation, ischemia, subendocardial vasculitis, and myocardial fibrosis, which are often missed by other imaging modalities, especially at an early stage of development. Plus, the presence of late gadolinium enhancement (LGE) has been linked to a worse cardiovascular prognosis in various patient populations. CMR is an important tool in the diagnosis and risk prediction for patients with sarcoidosis and could help narrow the gap between clinical and autopsy diagnosis of myocardial involvement in patients with SLE (40). CMR is surely helpful in the early detection of CVD risk, however, the considerable cost and limited availability have to be taken into account, as well as the need to perform clinical prospective trials in order to assess the specific parameters that affect CVD prognosis. Furthermore, CMR could be extremely valuable in some cases and can be considered for patients with new-onset heart failure, arrhythmia, for treatment evaluation, or if there is any mismatch between patient symptoms and routine non-invasive evaluation (41).

## Nuclear Imaging: Focus on Inflammation

Methods using nuclear imaging and CT, although promising, have the considerable disadvantage of ionizing radiation and thus, are difficult to justify as a preventative diagnostic method. In recent years, F-fluorodeoxyglucose-positron emission tomography/computed tomography (18 F-FDG-PET/CT) has shown its value in cardiac imaging for the diagnosis and follow-up of patients with inflammatory conditions of the heart like sarcoidosis, pacemaker infections, and endocarditis. It allows to assess vascular inflammation directly; it shows the quantification of 18F-2-deoxy-D-glucose uptake, and thus vascular inflammation, as well within the atheroma as overall in the arterial wall. An increased arterial FDG uptake has been shown to predict plaque expansion and rupture and thus leading to CV events (42, 43). As arterial inflammation is believed to represent one of the earlier and possibly reversible steps of atherosclerosis, and has been known to precede subsequent calcification, FDG-PET/CT has been increasingly used as a primary outcome in randomized controlled trials of anti-atherogenic drugs (44). PET/CT has been able to detect aortic vessel wall inflammation in RA patients without CVD symptoms (45) and has been proven to predict CVD better than the traditionally used Framingham risk stratification score (46). It has been demonstrated that patients with RA have significantly higher arterial FDG uptake compared with matched controls even after adjusting for atherosclerosis risk factors and statin therapy (47). Seraj et al. suggested that NaF-PET/CT might be even more effective at identifying increased molecular calcification in the wall of the abdominal aorta among patients with RA compared to FDG-PET/CT (48). Although there is a variety of compelling reasons that highlight the value of PET/CT imaging, it is considerably high in cost, not widely available, and will most likely not be included in routine preventative risk assessment in the clinical setting, but will still provide valuable information in the further research of CVD.

Biomarkers of CVD: the role of endothelial dysfunction Endothelial dysfunction is an early event in atherogenesis and has been known to precede the formation of plaques. There are several parameters that have been implicated as markers of endothelial dysfunction; among others, PWV and flow-mediated dilation have been evaluated thoroughly in atherosclerotic diseases. Additionally, biochemical parameters have emerged, such as compounds of the arginine metabolism asymmetric dimethylarginine (ADMA) or symmetric dimethylarginine (SDMA), and endothelial microparticles (EMP). These compounds mediate endothelial dysfunction through interaction with nitric oxide (NO) metabolism, vascular inflammation, and platelet function (49). In patients with systemic sclerosis (SSc) for example, ADMA and EMP might be involved in the development of microangiopathic changes and pulmonary arterial hypertension (50, 51). ADMA has been associated with a wide array of morphological and functional parameters of subclinical vascular disease in patients with autoimmune diseases (52). Significant correlations that have been established include between ADMA and carotid intima media thickness (53) as well as coronary flow reserve in patients with early RA (54) and psoriatic arthritis (55) or between ADMA and the detection of coronary calcium in patients with lupus erythematosus (56). Similarly, ADMA has been associated with arterial stiffness (57) and CVD events (58). In patients with systemic sclerosis and pulmonary hypertension, increased ADMA serum levels are negatively associated with exercise capacity (50, 59). This suggests that the NO pathway might play a significant role in the development of pulmonary vascular disease. Similarly, Thakkar et al. (60) could demonstrate that ADMA in combination with N-terminal pro hormone BNP (NT-proBNP) show an excellent sensitivity and specifity in the non-invasive detection of pulmonary arterial hypertension in patients with systemic sclerosis. Impaired endothelial function has been found for many other rheumatologic and autoimmune diseases; patients with SLE for example show impaired flow-mediated dilation, which in itself is considered as independent predictor of CV events (61). A growing body of evidence supports the hypothesis that chronic inflammation and immune dysregulation are pivotal in the development of atherosclerosis, which can in itself be considered as autoimmune disease (62). Activated T-lymphocytes expressing major histocompatibility complex (MHC) class II molecules with a pro-inflammatory T-helper (Th)-1 phenotype have been found in human atherosclerotic plaques; the activation of Th-1 responses contributes to a more aggressive progression of atherosclerosis (63). The adaptive immune system is targeted against self-antigens modified by a variety of biochemical factors such as oxidative stress and hypercholesterolemia. Atherosclerotic plaques have been found to express autoantigens, which are targeted by both IgM and IgG antibodies. Autoantigens, such as low-density lipoprotein (LDL), high density lipoprotein (HDL), and lesser-known autoantigens like stress-induced heat shock proteins (HSPs), beta-2-Glycoprotein 1 and oxidized hemoglobin have been associated with CVD, although their individual roles are still not entirely clear (64).

Considering autoimmune diseases, it has been found that synovium and atherosclerotic plaque show similarities in patients with RA, and thus, it has been proposed that common mechanisms might be at play in the accelerated atherosclerosis in RA patients (65). Likely as a consequence of chronic inflammation, RA patients show elevated LDL and HDL plasma levels. Similarly, patients with SLE typically have elevated levels of atherogenic lipoproteins and low levels of atheroprotective factors like paraoxonase 1 (66).

Still, there are limited studies examining the predictive value of vascular assessments on adverse cardiovascular outcomes in patients with rheumatologic diseases. Moreover, associations between disease-related inflammation and the vasculature are far from consistent (67). Even though inflammation seems to play a pivotal role in the mediation of CVD risk, the association between endothelial dysfunction and inflammation particularly in systemic inflammatory disorders stays controversial. In a prospective study with 201 RA patients and a follow-up of 6 years, classical CVD risk factors, such as hypertension, dyslipidemia and insulin resistance predicted vascular function and morphology better than disease-related inflammation (68). Another hallmark of autoimmune disorders is immune dysregulation, which in itself might increase the risk of CVD. Rheumatoid factor and antinuclear antibodies positive subjects have shown a higher risk of CVD events even after adjustment for the presence of rheumatic disease (63). Similarly, anti-CCP antibodies are associated with impaired endothelial function and myocardial involvement in patients with RA. Aforementioned ADMA could provide a promising link between endothelial dysfunction and autoimmune dysregulation as it has been shown to be associated with ds-DNA anti-SM, anti-RNP and anti-CCP among others (57).

High-density lipoproteins (HDL) are long known to have a pivotal role in the prevention of atherosclerosis. Altered levels of blood lipids and HDL have been described in a variety of autoimmune diseases, and the “lipid paradox,” where low lipid levels paradoxically correlate with increased CVD risk has been widely accepted, but the mechanisms are still not understood (69). One of the mechanisms might be reduced HDL functionality due to decreased enzymatic activity of the calcium-dependent esterase paraoxonase 1 (PON 1), which has been reported in these conditions. In RA, decreased serum PON 1 levels are associated with increased cIMT and plaques; thus, could be used as atherosclerosis prediction marker (70, 71). Although it has been discovered over 50 years ago, lipoprotein a (Lpa) has not gained importance up until the past 10 years, where it has shown to be an independent, genetic, and likely causal risk factor for CVD (72). Plus, it can be used for a broad spectrum of patients, including those with an LDL level of below 70 mg/dl. Its predictive value is considered higher than traditionally used markers, such as LDL, HDL and cholesterol (73).

In recent years, the role of autoantibodies in CVD has been explored; but although there are studies that suggest a link between humoral immune response and development of CVD, specific autoantibodies and their possible targets are yet to be elucidated. There seems to be a detrimental interplay between autoantibodies and lipid profiles. Autoantibodies targeting HDL have been shown to be associated with altered lipid profiles, and lipoprotein functionality (74–76). Interestingly, there seems to be a difference in anti-HDL levels among immune-driven diseases; Rodriguez-Carrio et al. (77) found the highest levels in systemic autoimmune rheumatic conditions and inflammatory bowel disease, whereas increased levels were not observed in organ-specific autoimmune diseases. Mixed connective tissue disease (MCTD) seems to exhibit an exceptionally high prevalence of anti-HDL positivity, and an association between anti-HDL antibodies and impaired PON1 activity in MCTD has been postulated (78). Hence, anti-HDL antibody levels might be a promising novel biomarker addressing the need for the identification of patients with lipoprotein dysfunction; anti-HDL levels can be measured through conventional, operator-independent and automatized laboratory techniques, thus making it a relatively cost-effective option.

Another potentially useful biomarker is Osteoprotegerin (OPG), which, as the name suggests, is traditionally implicated in bone remodeling but has been linked to CVD. OPG is produced by a variety of tissues and is a member of the tumor-necrosis factor (TNF) receptor family; it is known to be steadily released from vascular endothelial cells in response to inflammatory stimuli and thus might play a modulatory role in vascular injury and atherosclerosis (79, 80). Increased OPG levels have been related to a multitude of cardiometabolic alterations such as diabetes, obesity, hypertension, and metabolic syndrome. In patients with SLE, increased serum OPG has been associated with subclinical atherosclerosis (81), in RA elevated OPG levels correlated with cIMT and higher PWV (82). Hence, there is evidence that circulating OPG levels could be helpful in the identification of patients with subclinical atherosclerosis.

Another novel biomarker that has recently gained attention, endocan is a soluble dermatan sulfate proteoglycan released by the endothelium; it is known to be upregulated by multiple proinflammatory cytokines and proangiogenic factors and may be pro-inflammatory itself. In addition of being used as a surrogate marker of inflammation and endothelial dysfunction, it seems to be involved in the regulation of proliferative and neovascularization processes. Therefore, endocan has been proposed as a biomarker of endothelial dysfunction and pathological angiogenesis (78), thus suggesting its usefulness as a potential predictor of CV events and its utility as a biomarker has been increasingly explored for a variety of patient populations (83). High endocan levels were detected in autoimmune diseases as psoriasis (84), Behçet's disease (85), SLE (86), and SSc (87).

As liquid biopsies and new molecular biology techniques are used more frequently, a wide array of novel potential biomarkers has emerged on the horizon. A selection of the markers presented in this review can be found in Table 1. However, there are new and exciting markers emerging constantly. Adiponectin, for example, has been proposed as an early marker of atherosclerosis in asymptomatic type 1 diabetes mellitus patients (88). EMPs, microRNAs, ANGPTL8, CTRP9, and Galectin-3 among others have been studied in CAD patients (78), it is unclear, to which extent these findings could be applicable to patients with immune-driven conditions. Still, our knowledge of the complex interplay of the pathophysiology of endothelial dysfunction and atherosclerosis is better understood; the future will show, which of these novel markers will prove their value.

**Table 1:** Recently identified potential biomarkers of inflammation and endothelial dysfunction in rheumatic diseases.

**Biomarker**	**Implications or considerations**	**References**
Paraoxonase 1	Decreased serum PON 1 levels are associated with increased cIMT and plaques in RA	(55)
Lipoprotein a	Independent, genetic risk factor for CVD even in populations with low to normal LDL	(57)
Anti-HDL	Highest levels in systemic autoimmune diseases	(61)
Osteoprotegerin	Increased OPG levels have been related to cardiometabolic alterations such as diabetes, obesity, hypertension, and metabolic syndrome. In patients with SLE, increased serum OPG has been associated with subclinical atherosclerosis, in RA elevated OPG levels correlated with cIMT and higher PWV	(65, 66)
Endocan	Has been proposed as a biomarker of endothelial dysfunction and pathological angiogenesis. High endocan levels were detected in autoimmune diseases as psoriasis, Behçet's disease, SLE, and SSc	(63, 67–71)

## Artificial Intelligence: Transforming the Possibilities of Medical Imaging

On the quest for improving and optimizing preventative diagnostics, Artificial Intelligence (AI) has recently emerged as a novel tool with the potential to radically change the way we interpret data and make clinical decisions. With increasing data volume and complexity, AI techniques such as machine learning and deep learning can be an invaluable tool to extract relevant information (89). Machine learning is a subfield of AI used to “teach” computers to analyze vast datasets quickly and efficiently; making it possible to identify patterns on new data that match with existing data. Deep learning is a machine learning technique characterized by its use of neural networks, which learn through experience, read data, can build hierarchical architectures, and provide more advanced input-output levels. Deep learning can work with more complex nonlinear patterns and is gaining popularity in the medical research field, as data is steadily increasing in volume and complexity. Deep learning techniques are already playing a pivotal role in tech companies, for example in the field of speech recognition in Apple's Siri and Amazon's Alexa, and Facebook image recognition programs (90).

Machine learning based predictive models might provide more accurate information on CVD risk: In a prospective cohort study using routine clinical data of 378,256 UK primary care patients, Weng et al. (91) were able to show that machine learning algorithms outperform established risk prediction approaches at predicting the absolute number of CVD cases correctly. Likewise, Jamthikar et al. (92) found machine learning based CVD/stroke risk calculators to be superior in terms of 10-year CVD/stroke risk prediction, compared to t13 different types of statistically derived risk calculators.

The advantage of AI techniques in the medical field are numerous; in echocardiography, inter- and intraoperator variability has been shown to be reduced and it is possible to detect additional predictive information which is too subtle for the human eye to see (93). This makes the application of AI especially compelling in the early detection of CV changes. A new up-and-coming option for AI could be its use in cardiac CT: the association between cardiac CT and machine learning algorithms has shown a promising chance in clinical practice to detect functional information beyond atherosclerotic plaque characterization (94).

It has been shown over and over again that risk stratification methods aimed at the general population fall short in the assessment of patients with immune-mediated autinflammatory diseases. AI techniques might help bridge this gap and help clinicians to tailor predictive medicine to the individual patient (95).

Although the research in this field is promising, data focusing specifically on patients with immune-mediated inflammatory diseases is scarce. Additional studies are needed to evaluate the potential of AI as a tool for more personalized and thus effective decision-making.

## Discussion

CVD has long been recognized as a major cause of premature morbidity and mortality among patients with immune-driven conditions. Although we gain a growing understanding of the mechanisms that fuel the vicious circle of inflammation and atherosclerosis, there is still a lack of comprehensive approaches of risk stratification, preventative care, and treatment options. A multitude of surrogate parameters have emerged to help pinpoint patients most at risk at an early stage. PWV and AIx have been widely used in the scientific community to assess CVD risk in subclinical populations, but are not yet routinely used in a clinical setting. There are numerous studies that show that PWV, as well as AIx, are predictive for CVD events and all-cause mortality in asymptomatic populations. Given the fact that modern devices are reasonably portable, relatively easy to use, time-efficient and non-invasive, their integration in every day routine rheumatology practice could improve CVD screening of patients with systemic inflammatory diseases. Carotid ultrasound, on the other hand, has been a valuable tool in the detection of asymptomatic patients with CVD, and can be supplemented by additional imaging methods such as CMR; PET/CT scan can be considered in unclear cases. The advent of AI techniques in modern medicine is an up-and-coming tool which can be useful in the interpretation of vast data volumes and complexity. Novel biomarkers like PON1, Osteoprotegerin, or Endocan have emerged with promising data, but still need to be examined further in relation to diagnostic value and if they can be applied to different population groups. However, it is difficult to draw specific conclusions from the current evidence regarding the mechanisms through which those parameters could be interpreted about their possible prognostic value. Although there is evidence that combining several methods leads to a higher accuracy, the optimal combinations for diagnosis or prognosis still need to be defined. A holistic, comprehensive approach seems to be the most optimal way to pinpoint patients most at risk for CVD. More longitudinal studies with a variety of populations are needed to further describe and assess their prognostic value as well as the best way to employ them in daily clinical practice. Still, we already see promising evidence which might change the way we can identify patients at risk for CVD which would have otherwise been stratified incorrectly with traditional methods. Early atherosclerotic lesions are reversible, and the incorporation of diagnostic methods like PWV, AIx, newer imaging techniques and novel biomarkers could help establish an early diagnosis and prevent the occurrence of CV events early on and thus, facilitate a better outcome and quality of life.

## Author Contributions

AM acquired and analyzed the data and wrote the first draft of the manuscript. AS, LC and AM drafted the manuscript. AS and LC revised the work critically for important intellectual content. KT conceived of the work, drafted the manuscript, and revised it critically for important intellectual content. All authors have read and approved the final version of the manuscript.

## Conflict of Interest

The authors declare that the research was conducted in the absence of any commercial or financial relationships that could be construed as a potential conflict of interest.

## Publisher's Note

All claims expressed in this article are solely those of the authors and do not necessarily represent those of their affiliated organizations, or those of the publisher, the editors and the reviewers. Any product that may be evaluated in this article, or claim that may be made by its manufacturer, is not guaranteed or endorsed by the publisher.
